# Association of Variants in Innate Immune Genes *TLR4* and *TLR5* with Reproductive and Milk Production Traits in Czech Simmental Cattle

**DOI:** 10.3390/genes15010024

**Published:** 2023-12-23

**Authors:** Karel Novák, Terezie Valčíková, Kalifa Samaké, Marek Bjelka

**Affiliations:** 1 Department of Genetics and Breeding, Institute of Animal Science, Přátelství 815, 104 00 Prague-Uhříněves, Czech Republic; 2 Department of Genetics, Czech University of Life Sciences, Kamýcká 129, 165 06 Prague, Czech Republic; teschiro@gmail.com; 3 Department of Genetics and Microbiology, Viničná 7, Charles University, 128 43 Prague, Czech Republic; kalise.study@gmail.com; 4 Breeding Company CHD Impuls, 592 55 Bohdalec, Czech Republic; mbjelka@chdi.cz

**Keywords:** cattle, genotyping, immunity, inflammation, mammary gland, parturition, toll-like receptors

## Abstract

Bovine genes *TLR4* and *TLR5*, which encode antibacterial toll-like receptors, were screened for polymorphisms in Czech Red Pied (Czech Simmental) cattle to identify variants associated with reproduction, udder health, and milk production traits. Variants were discovered by hybrid resequencing of 164 bulls using HiSeq X-Ten and PacBio technologies and then individually genotyped. Nominal *p*-values < 0.05 for associations were detected in 18 combinations between 14 polymorphisms and 15 traits using one-way analysis of variance (ANOVA). The *TLR4* variants g.610C>T (rs43578094) and g.10310T>G (rs8193072) in reference AC000135.1 were strictly associated with the index of early reproductive disorders and maternal calving ease, respectively, at false discovery rate (FDR) < 0.05. A highly permissive false discovery rate cutoff of 0.6 separated seventeen combinations in both genes comprising eight positives. In the case of the *TLR4* variant g.9422T>C (rs8193060), indications were obtained for the association with as many as four reproductive traits: incidence of cystic ovaries, early reproductive disorders, calving ease, and production longevity. The permissive FDR interpretation for the *TLR5* data indicated associations with cyst incidence and early reproduction disorders with maternal calving ease. Moreover, three *TLR5* polymorphisms correlated with milk production traits. The discrepancy of the observed associations with the predicted impacts of the SNPs on protein function points to the role of haplotypes. Nevertheless, this question should be resolved on a larger scale. The observed associations are endorsed by independent evidence from the published functional roles in other species and by the published QTL mapping data.

## 1. Introduction

Targeted genotyping of variations in causal genes for health traits can be considered a complement to the generally adopted genomic selection in cattle [1]. Its use might lead to faster improvements in the population structure for the desired gene variants. Accordingly, breeding for health traits in cattle is an alternative to veterinary measures [2].

The innate immune *TLR* genes are considered to be prospective targets for breeding [3] since they code for a series of toll-like receptors that play a key role in the recognition of bacterial, fungal, and viral determinants. In cattle, the products of the *TLR1*, -*2*, -*4*, and -*6* genes recognize polysaccharide or glycoprotein ligands released from the cell walls of Gram-negative and Gram-positive bacteria, while the *TLR5* product recognizes the flagellin protein from bacterial flagella [4]. Natural variation in the bovine *TLR* genes has been documented in the panel of world cattle breeds [5,6,7], and an association between this polymorphism and health traits has been reported [7,8,9].

Their role has also been demonstrated in many immune and nonimmune traits, both on the molecular and organismal levels [10]. For instance, the predicted functional change encoded by c.2021C>T affecting the transmembrane region of the *TLR4* product was associated with a shift in somatic cell count in milk as an indicator of inflammation [11,12].

Recently, an effect on the parturition in cattle has been demonstrated in the work by the authors [13]. Consequently, the aim of the present work was to verify whether this finding can be extended to the other two antibacterial toll-like receptor genes—*TLR4* and *TLR5*. The narrow objective of the present work was to explore the diversity of the *TLR4* and *TLR5* innate immune genes in the Czech Red Pied (CRP) cattle breed and characterize the associations between their variation and chosen traits encompassing milk production, udder health, and reproduction. Polymorphism screening at the population level was performed by resequencing a pooled DNA sample with two different technologies for next-generation sequencing (NGS) to take advantage of the hybrid approach. The variants of both genes discovered in the population were correlated with the estimated breeding values (EBVs) for phenotypic traits. The observed associations were interpreted in light of the computer-predicted impacts of gene variants and the context of reports from model species and previous mapping of QTLs for reproductive traits in other cattle populations.

## 2. Materials and Methods

### 2.1. Animals

The DNA collection used originated from 164 CRP cattle bulls from the portfolio of the main breeding enterprise for this breed, CHD Impuls (Bohdalec, Czech Republic). Czech Red Pied cattle are a local Simmental breed that was formed from imported Simmental cattle during the nineteenth century. The herd today represents more than one-half of cattle in the Czech Republic and the breed is also exported worldwide. Although CRP is considered a dual-purpose breed, its dairy production is dominant, with a breeding goal of 7500–8500 kg milk/year. The breeding goal for bull daily gain is above 1300 g. To increase the basis for husbandry, the bulls of the related breeds Fleckvieh and Montbélliard were admitted for registration in the breed book in 2000. Consequently, the convergence of gene pools with the major Simmental breeds is taking place, although the structure of the breeding indices and breeding goals are defined separately for CRP and the population is still considered distinct.

### 2.2. DNA Samples

Genomic DNA was prepared from the cryopreserved insemination doses using a MagSep Tissue Kit processed by an EpMotion 5073 pipetting station (Eppendorf, Hamburg, Germany). Isolated DNA was checked for purity by UV absorbance spectroscopy and on-plate fluorimetry using SYBR Gold stain (Biotium, Fremont, CA, USA). A normalized set containing 20 ng/µL of genomic DNA from each animal was prepared as the input for serial PCR amplification. The purity provided by the MagSep kit met the requirements of both the PCR amplification and subsequent primer-extension reactions.

### 2.3. Sequencing with PacBio Technology

The coding sequences and flanking regions of *TLR4* and *TLR5* were PCR-amplified with primers and under conditions (Supplementary Table S1) that were adapted from previous studies [14,15,16]. The two large intronic regions of *TLR4* were left out. The amplification was performed on a pooled gDNA sample comprising DNA from all 164 animals. The amplicons were sequenced with PacBio technology (Pacific Biosciences, Menlo Park, CA, USA) using P4-C2 chemistry. The library for single-molecule real-time (SMRT) sequencing was prepared in the GATC-Biotech core laboratory (Constance, Germany). The circular consensus sequencing (CCS) protocol was applied to obtain one 120 min data collection record on the PacBio RS II machine. On average, 6 reads were obtained per haploid amplicon per animal for the starting set of 164 animals. However, statistical noise prevented the reliable detection of variants with frequencies below 0.02.

### 2.4. Low-Coverage Whole-Genome Sequencing

Whole-genome sequencing of a pooled population sample of gDNA was applied for the validation of PacBio technology-detected polymorphisms using the hybrid strategy [17]. The HiSeq sequencing library (Illumina, San Diego, CA, USA) was prepared in the laboratory of Novogene (London, UK). X-ten technology was used to perform two rounds of paired-end (2 × 150 bp) sequencing. The obtained 60× coverage was sufficient for the reliable detection of polymorphisms above a frequency of 0.05 with 95% efficiency.

### 2.5. Processing of Sequencing Data

The PacBio reads were mapped to the reference sequences AC000135.1 and EU006635 (NCBI nucleotide database) for *TLR4* and *TLR5*, respectively, following the source publications describing these cattle genes [14,15] using the Geneious program package (Biomatters, Auckland, New Zealand). The positions of the found variants were lifted over to the coordinates of the UMD_3.1.1 assembly of the cattle genome. The HiSeq reads were mapped directly to the UMD_3.1.1 reference sequence at a minimum mapping quality = 50 using the Geneious Mapper algorithm implemented in the Geneious program. Structural variants were detected in the assembly at a maximum *p*-value of 0.01 and a minimum number of reads = 3. The variants were filtered for SNP clusters formed from the misaligned reads, where clusters were defined as more than 3 variants in a 25 nt stretch. The filtered variants were validated by the mutual comparison of the results obtained with the two sequencing platforms. The presence of the variant in the EBI Variation Archive at https://www.ebi.ac.uk/eva/?Home (accessed on 19 December 2023) was considered as an additional independent confirmation.

### 2.6. Mutation Effect Prediction

The SNPs revealed by hybrid sequencing were evaluated for their predicted impact with the Variant Effect Predictor (VEP) application (https://www.ensembl.org/info/docs/tools/vep/index.html, accessed on 19 December 2023). In parallel, functional changes to the proteins were estimated by their SIFT (Sorting Intolerant from Tolerant algorithm) value according to Sim et al. [18].

### 2.7. Genotyping

The validated SNPs were genotyped in individual animals using primer extension reactions performed with the commercial SNaPshot multiplex system according to the instructions of the manufacturer (Thermo Fisher Scientific, Carlsbad, CA, USA). The same amplicons as those used as the input for NGS were used as a substrate for genotyping. The SNaPshot reactions were multiplexed by 4 or 5 by adding poly-T extensions to the 5′-end, as indicated in the list of detected SNPs in Table 1. The primers of the same multiplex were staggered by five nucleotides to accomplish simultaneous separation by the ABI PRISM 3100 capillary sequencer (Applied Biosystems, Carlsbad, CA, USA). The peaks resulting from fragment resolution were identified visually.

### 2.8. Phenotypic Traits

The EBVs of the bulls in the cohort, as determined from the data on the daughter cow population, are suitable as the input in association studies, as demonstrated by Richardson et al. [19]. The EBVs for milk production, udder health, and reproductive traits were downloaded from the central cattle evaluation system database (as of August 2018) maintained for the Czech Republic by the breeding data database Plemdat (Hradišťko). The production characteristics included milk fat percentage (FP), milk fat yield (FY), milk protein percentage (PP), milk protein yield (PY), and milk yield (MY). The udder health-related traits included somatic cell score (SCS), udder health index (UHI), milkability (MLA), and lactation persistency (PER). The incidence of cystic ovaries (CYS), early reproductive disorders (ERDs), calving ease (CE), maternal calving ease (CM), length of productive life (PL), and the calf vitality index (CVI) formed a group of reproductive traits. The CVI value is calculated from the breeding values for stillborn calves and survival rates for 30 days, 10 months (bulls), or 15 months (heifers) with weights of 0.52, 0.24, and 0.12, respectively.

### 2.9. Statistical Tests

The allelic frequencies based on individual genotyping data were matched with the frequencies derived from the NGS read representation (averaged for the two technologies used). The NGS-based frequencies and genotyping-based frequencies were partially published elsewhere [16,20]. The Hardy–Weinberg (H-W) equilibrium of the genotype classes was tested with the χ^2^ criterion. The strength of a genotype-to-phenotype association was characterized by a one-factor analysis of variance (ANOVA). The Benjamini–Hochberg procedure [21] was applied to the multiple comparisons of all combinations at varying false discovery rate (FDR) values. In parallel, the average values for phenotypic trait EBVs were pairwise compared across the genotypic classes using the two-tailed *t*-test.

The linearity of the bull EBVs and potential allelic Interactions were tested using the regression coefficients in the linear model prepared with the REML algorithm in the WOMBAT software [22], downloaded from http://didgeridoo.une.edu.au/km/wombat.php, last accession 12 December 2023. The potential bias of the association values due to sire relatedness was estimated in REML by the inclusion of the pedigree matrix covering two generations of ancestors.

### 2.10. Quantitative Trait Loci

The positions of quantitative trait loci (QTL) for cattle reproductive traits were downloaded from the animal QTL database at https://www.animalgenome.org/cgi-bin/QTLdb/BT/ (accessed on 19 December 2023), which is maintained by the National Animal Genome Research Support program of the United States Department of Agriculture.

## 3. Results

### 3.1. Diversity Detected

A list of the seven *TLR4* and seven *TLR5* variants that were detected in the CRP population with NGS and subsequently genotyped for the association study is presented in Table 1 along with their basic characteristics.

The frequencies in individually determined SNPs mostly corresponded to those derived from the NGS read occurrence (Table 2), except for g.7999A>G of *TLR4* and g.619T>G with g.1736C>T of *TLR5*. However, the allelic frequency obtained by individual genotyping was considered to be decisive for the subsequent calculations. Departure from the Hardy–Weinberg equilibrium was observed only in the SNP g.245G>C of *TLR4* and g.545T>C of *TLR5* (Table 2). Nevertheless, the ANOVA test for allele association is independent of a systemic deviation from the expected ratio, which allows it to be applied in this case.

### 3.2. Variant Effect Prediction

The predicted effects of the genotyped gene variants on protein function as calculated by the VEP and SIFT programs are listed in Table 1. All genotyped polymorphisms were in noncoding regions or were synonymous. Consistently, the predicted effects were graded as negligible or low. Only the g.245G>C SNP, which is located in the 5′ untranslated region of *TLR4*, has been predicted to exert an effect on *TLR4* gene expression [23,24].

### 3.3. Association between Genotypes and Trait Values

**Figure 1:**
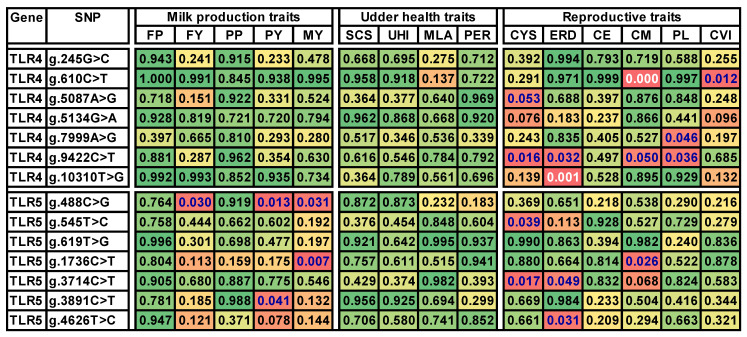
Significance values for a one-factorial ANOVA of the associations of *TLR4* and *TLR5* genotypes with the breeding values for milk production, udder health, and reproductive traits. Significance coding: nonsignificant nominal *p*-values above 0.05 are on green background turning to yellow, *p*-values below 0.05 are already marked in red. In parallel, the significance of the P_FDR_ is marked with the letter color. The trait × SNP combinations significant at FDR < 0.6 are in blue letters, and combinations significant at FDR < 0.1 are in white letters. Abbreviations: FP, milk fat percentage; FY, milk fat yield; PP, milk protein percentage; PY, milk protein yield; MY, milk yield; SCS, somatic cell score; UHI, udder health index; MLA, milkability; PER, lactation persistency; CYS, incidence of cystic ovaries; ERDs, early reproductive disorders; CE, calving ease; CM, maternal calving ease; PL, length of productive life; CVI, calf vitality index.

The results of the association test between the genotype classes and the trait values are presented in Figure 1, where the nominal *p*-values are visualized in the background color scale. Altogether, nominal *p*-values below 0.05 were observed in 18 out of the 210 total combinations of the 14 tested SNPs and 15 traits. Unexpectedly, variants of both *TLR4* and *TLR5* were predominantly associated with the group of reproductive traits.

However, precautions in the result interpretation are necessary due to the possibility of false positive combinations originating from multiple comparisons. In order to estimate the role of false positive results (Figure 1), the Benjamini–Hochberg procedure [25] was applied. At the selected value of FDR < 0.1, the associations in the two traits vs. SNP combinations were significant, namely maternal calving ease × g.610C>T and the index of early reproductive disorders × g.10310T>G. Only the former combination was significant at a stricter value of FDR < 0.05.

A permissive cutoff value of 0.6 for FDR enabled the separation of a group of additional 17 candidate trait × SNP combinations. This group of combinations is assumed to contain additional six true positives. In the case of the *TLR4* variant g.9422T>C, indications were obtained for the association with four different reproductive traits: cystic ovary incidence, early reproductive disorders, calving ease, and production longevity. In addition, g.610C>T was presumably associated with the calf vitality index, g.5087A>G with cyst incidence, and g.7999A>G with production longevity, a trait also including reproductive traits.

In *TLR5*, the high FDR interpretation suggested an association with cystic ovary incidence in g.545T>C and g.3714C>T, early reproductive disorders in g.3714C>T and g.4626T>C, and maternal calving ease in g.1736C>T.

In the group of milk production traits, only some polymorphisms in *TLR5*, namely g.488C>G, g.1736C>T, and g.3891C>T, were presumably in association with the permissive FDR rate. In contrast to the expectation based on the published studies, no correlation of the variation in the *TLR4* or *TLR5* genes with udder health-related characteristics was observed.

Generally, the group of reproductive traits demonstrated a higher statistical resolution in the current study. The reason might be a more precise recording of reproductive data compared to the other traits.

### 3.4. Additional Statistical Tests

The conclusions from the ANOVA test were independently confirmed by calculating the significance of the pairwise differences according to the *t*-test for genotypes (Supplementary Table S2). The table also contains the average values for the traits in genotypic classes. The *t*-test turned out to be more sensitive than the one-way ANOVA since four significant differences were detected in addition to eighteen associations revealed by nominal *p*-values in ANOVA, including g.5087A>G × FY and g.5134G>A × CYS in *TLR4* and g.545C>G × ERD with g.3714C>T × CM in *TLR5*. Consistently, the ANOVA test results for these SNPs approached the 0.05 significance level from the top (Figure 1). The match between the genotype mean comparisons by the *t*-test and ANOVA can be treated as an additional confirmation of significant combinations.

The assumptions of additivity, dominance/recessivity, and full heterosis were tested using the REML procedure. The pattern of *p*-values for genotype × trait combinations basically followed the ANOVA results, as indicated by the inter-matrix correlation coefficient of 0.753 for the additive model. On the other hand, no radical improvement in the test resolution power was observed. Furthermore, the assumptions about the dominance or full heterosis lead to improved *p*-values compared to the additive model. Therefore, the ANOVA results were the first choice for subsequent interpretation; moreover, they are free of assumptions about the mechanism of gene action [26].

The animal relatedness bias was excluded by comparing the results before and after the inclusion of the pedigree matrix for two generations of ancestors in the REML procedure. The changes to the *p*-values were negligible; therefore, the effect of relatedness was not considered in the ANOVA processing.

## 4. Discussion

### 4.1. Aberrant Hardy–Weinberg Equilibrium

Although the behavior of the monitored markers generally corresponded to the expectation, the departures of the g.245G>C SNP of *TLR4* and g.545T>C of *TLR5* from the H-W equilibrium might indicate homozygous lethality. However, this explanation is not consistent with the localization of these SNPs in the 5′ untranslated and 5′ flanking regions, respectively, with weak predicted effects. Moreover, loss-of-function mutations in the antibacterial *TLR* genes with dramatic effects have not yet been reported for cattle. Even though the SNPs in *TLR4* created stop codons at amino acid positions 534 and 746 and caused lesions in the extracellular leucine-rich region (LRR) and the conserved toll/interleukin-1 receptor homology (TIR) region, respectively, they had no consequences for vitality in Holstein cattle [9].

### 4.2. Associations with Phenotypic Traits

Only g.245G>C in *TLR4* of the genotyped polymorphisms is predicted to have an effect on gene expression due to the disturbed binding of transcription factors [22,24]. Although this prediction is consistent with the previously reported association with somatic cell score in the Holstein population [9], no correlation was detected under our conditions. Similarly, no relation between this *TLR4* polymorphism and somatic cell count was detected in another study on the Holstein population [24].

The absence of an association of the remaining *TLR4* functional polymorphisms with milk production and udder health traits in our work can be explained by the moderate impacts predicted. Analogously, Opsal et al. [27] failed to detect any significant association between *TLR2* and *TLR4* regions and mastitis in Norwegian red cattle. Nevertheless, significant associations with milk production traits have been observed in some SNPs in *TLR4* not included in this study, e.g., c.2021C>T (g.9787C>T), according to previous reports [12,22].

On the other hand, the polymorphism 9422T>C of *TLR4*, albeit synonymous and predicted to be neutral, demonstrated indications of associations with four reproductive traits in our study. Furthermore, the putative associations and shared phenotypic patterns of the neutral mutations in *TLR4*, namely g.610, g.5087, g.5134, g.7999, g.9422, and g.10310, also suggest an unidentified causal polymorphism. The genotyped SNPs are probably not the true causal variants and could just be in linkage disequilibrium with the causal SNPs in extended haplotypes covering the regulatory regions [28]. The haplotype structure in the coding regions of *TLR4* and *TLR5* in the CRP population was described by Novák et al. [29] and Samaké and Novák [20].

The association of *TLR*s with the trait of calving ease in cattle has already been reported by Mullen et al. [30] for the g.5087A>G SNP in *TLR4* (rs8193046). This polymorphism was primarily associated with infectious bovine keratoconjunctivitis (IBK) caused by *Moraxella bovis*. The mutation is intronic without a predicted effect, similar to most of the SNPs in our study. The association of this SNP with calving ease was not detectable in our work, although the nominal *p*-value was low (0.053) with respect to the incidence of cystic ovaries. Obviously, the health trait associations of g.5087A>G should be also ascribed to its diagnostic role for the haplotype structure.

When considered in detail, allele g.5087A was associated with g.9422C and g.9787C in haplotype A2, according to the traditional classification of the *TLR4* haplotypes [14,20], while the complementary allele g.5087G was paralleled with the bases g.9422T and g.9787T in haplotypes A5 and B1, respectively. Consistently with this assumption, the g.5087A>G-linked mutations g.9422T>C (rs8193060), albeit synonymous, were associated with reproductive traits, including maternal calving ease, in our work. Another SNP linked to g.5087A>G in these haplotypes, g.9787C>T (c.2021) [14,20], is known to affect signal transfer due to the functionally relevant amino acid change (Thr674Ile) in the transmembrane region of the molecule [14]. This mutation has been traditionally studied due to its demonstrated association with somatic cell score [11,12].

Recently, El-Domany et al. [31] detected the association of an AluI polymorphism in *TLR4* with the reproductive traits including the age at first freshening, calving interval, number of services per conception, ovarian rebound, and days open in a Holstein population. However, the used AluI pattern is located in the central part of the first intron region and cannot be matched with the SNPs described in this study.

In gene *TLR5*, tentative associations of the neutral SNPs with reproductive traits were observed as well. Mutually similar association patterns for the early reproductive traits observed in SNPs g.545, g.3714, and g.4628 might reflect the absolute linkage of g.545 and g.3714 consistently with two major *TLR5* haplotypes [20].

### 4.3. Association Patterns Related to the TLR4 and -5 Mechanisms of Action

The different molecular functions of the TLR4 and TLR5 proteins should result in different association patterns with phenotypic traits included in this study. The TLR4 receptor recognizes lipopolysaccharide and peptidoglycan bacterial cell wall components, while the *TLR5* product recognizes a molecule of the flagellin protein that indicates the presence of bacterial flagella. For example, the observed association of the *TLR4* variants with calf survival might reflect the TLR4 role in resistance to the major respiratory and gut infections of calves.

The associations of the *TLR* variants with the calving ease traits might also be primarily mediated by uterine infections [32,33]. Alternatively, the variability in *TLR* gene products can affect parturition directly since the TLR4 protein, along with TLR2 and its interactors TLR1 and TLR6, participates in the inflammatory response and myometrial signalling before parturition via the MyD88/TRAF6/NF-κB pathway [34]. The central role of TLR4 in the timing of parturition and its effect on perinatal viability has been demonstrated in mice using the TLR4-deficient genotype [35]. Convincingly, preterm birth induced by bacterial LPS administration in mice is prevented in the *TLR4* knockout line [36]. The central role of TLR4 in perinatal signalling has made it a target for the development of drugs based on rosiglitazone intended to reduce the risk of premature birth [37].

Additional yet unknown nonimmune mechanisms might mediate the effect of *TLR* variation on early reproductive traits, such as cystic ovary incidence and early reproductive disorders. Almost all *TLR*s are expressed in different patterns in the ovary during the estrous cycle, and *TLR*s also participate in sperm capacitation in the female reproductive tract [10]. A direct link between *TLR* activity and fertilization has been reported in a mouse model [38].

### 4.4. Context of the Known QTLs

Notably, a match between the location of *TLR4* on chromosome 8, where the genotyped *TLR4* markers span from 108,829,143 to 108,839,208 bp, with QTL #43837 for calving ease at 108.8 Mbp, corresponding to 122.70 cM and SNP with rs43053569 [39], is remarkable and can be treated as an independent validation of the assumed effect of the *TLR4* variants. However, an accidental correspondence cannot be fully excluded since #43837 is only one of 32 QTLs for calving ease located on chromosome 8 according to the bovine QTL database.

Similarly, a good match between the location of *TLR5* on chromosome 16 (with the span of monitored SNPs from 27,303,645 to 27,307,783 nt) and QTL #48258 for calving ease with a maximum at 27.5 Mbp, i.e., at 33.56 cM and rs109206036 [39], endorses the hypothesized *TLR5* effect on calving. In spite of the apparent coincidence, QTL #48258 is only one of 59 calving ease associations located on chromosome 16. Therefore, the final conclusion on the matches of *TLR4* and *TLR5* with candidate QTLs depends on the future identification of the QTL causal variants.

In a published genome-wide association study of calving performance in Holstein, Charolais, and Limousine breeds, candidate gene locations on chromosomes 2, 4, and 18 and the putative causal missense variants in the *SIGLEC12*, *CTU1*, and *ZNF615* genes were reported [40]. Like in our study, a strong signal has been observed for an intronic variant of the gene *PCLO* on chromosome 4, suggesting the causal role of a yet unidentified linked polymorphism.

### 4.5. Impact on Breeding

Although the statistical evaluation of associations based on the bulls’ EBV seems to be sufficiently sensitive and reliable, as demonstrated in the work by Richardson et al. [19], it is desirable to extend the study to the primary data from a wider cow population. A collection of 1200 heifers of the breed is being genotyped for the *TLR* variants in order to further precise the conclusions from the primary association study.

Nevertheless, the findings are applicable already now in the environment of the breed. A versatile genotyping tool covering the SNPs with effect on reproductive traits is under development. Additional information might be included in the current breeding index, albeit with a low weight, in order to enrich it with fitness traits.

## 5. Conclusions

In the population of Czech Red Pied cattle, the polymorphism in *TLR4* and *TLR5* tended to be associated with reproductive traits, while only some *TLR5* variants were associated with milk production. The causative variants are probably not located in the regions covered by targeted genotyping but are in linkage with them. The implied effects of the *TLR4* and *TLR5* polymorphisms can be considered an extension of the association of the *TLR1*, *-2,* and *-6* complexes with reproductive traits reported previously [20]. Additional experiments discriminating between the two mechanisms of action, either by infection or nonimmune signalling in fertilization and parturition, are necessary.

## Figures and Tables

**Table 1:** Basic characteristics of genotyped polymorphisms of *TLR4* and *TLR5* in the representative bull population of Czech Red Pied cattle.

SNP Identifier	Chromosome/ /Position	Substitution ^a^	PCR Fragment	Extension Primer		Multiplex Part	SNP Type + Effect Prediction
				Primer Name	Sequence 5′→3′		
*TLR4*							
rs29017188	8_108829143	g.245G>C	4_1	T245F	CTTCTTCTTCCTCTAACTTCCCCTC	A1	5′-UTR, expression
rs43578094	8_108829508	g.610C>T	4_1	T610R	(T5)GGGCCCAGCACAGGGAAACTGAGCA	A2	intron, modifier
rs8193046	8_108833985	g.5087A>G	4_2	T087F	(T10)GCTAAGGTGCATGCAGGAAGACACC	A3	intron, modifier
rs8193047	8_108834032	g.5134G>A	4_2	T134R	(T13)GATTTTGTAGAGATTCAGCTCCATGCA	A4	synonymous, low
rs43578100	8_108836897	g.7999A>G	4_3	T999F	(T21)GGTTTCCTATTCAGCAGAAATATT	A5	intron, modifier
rs8193060	8_108838320	g.9422C>T	4_4	T422F	ACTCGCTCCGGATCCTAGACTGCAG	B1	synonymous, low
rs8193072	8_108839208	g.10310T>G	4_5	T010F	(T25)CCACCTGAGGAGGAGAATCCCCTGA	C4	3′-UTR, low
*TLR5*							
	16_27307783	g.488C>G	5_1	T488F	(T6)CCAGGGATGAAACCCRTGTCTCCTG	B2	upstream, modifier
ss73689429	16_27307726	g.545T>C	5_1	T545F	(T10)CCAGGGAAGTCTTGCTGGCCTACTG	B3	upstream, modifier
ss73689429	16_27307652	g.619T>G	5_2	T619R	CCACAGCACCTTTGAGGCTGTGAC	C1	upstream, modifier
ss73689443	16_27306535	g.1736C>T	5_3	T736F	(T2)GTACTTACAAYCATGCTTGCTATTTTT	C2	upstream, modifier
rs55617187	16_27304557	g.3714C>T	5_4	T714R	(T15)GATTGAGCCAATGGATAAAAGCACT	B4	synonymous, low
rs55617251	16_27304380	g.3891C>T	5_4	T891R	(T18)CACGAGGAACAGAGTCAAGGTGACAGT	B5	synonymous, low
rs55617288	16_27303645	g.4626T>C	5_5	T626R	(T9)GGGGTCGCAAAGAGTAGGACATGACC	C3	downstream, modifier

^a^ SNP positions were determined in the AC000135.1 and EU006635 reference sequences for *TLR4* and *TLR5*, respectively.

**Table 2:** Frequencies and Hardy–Weinberg equilibrium of the monitored SNPs in *TLR4* and *TLR5* of the Czech Red Pied cattle population.

Gene	SNP ^a^	Number of Bulls	Genotype Frequencies Observed			Allelic Frequencies		χ^2^	WT Allele Frequency in NGS	
			Homozygotes 1	Heterozygotes	Homozygotes 2	*p*	q		WT Read Frequency ^c^	% of *p*
TLR4	g.245G>C	81	0.086	0.630	0.284	0.401	0.599	7.804 ^b^	0.457	113.9
TLR4	g.610C>T	72	0.861	0.139	0.000	0.931	0.069	0.401	0.937	100.7
TLR4	g.5087A>G	73	0.329	0.493	0.178	0.575	0.425	0.006	0.746	129.7
TLR4	g.5134G>A	76	0.724	0.250	0.026	0.849	0.151	0.054	0.694	81.8
TLR4	g.7999A>G	80	0.163	0.600	0.238	0.463	0.538	3.421	0.754	163.0
TLR4	g.9422C>T	66	0.182	0.348	0.470	0.356	0.644	3.803	0.231	64.9
TLR4	g.10310T>G	84	0.679	0.321	0.000	0.839	0.161	3.080	0.625	74.5
TLR5	g.488C>G	81	0.185	0.395	0.420	0.383	0.617	2.175	0.385	100.6
TLR5	g.545T>C	70	0.414	0.357	0.229	0.593	0.407	4.739 ^b^	0.500	84.3
TLR5	g.619T>G	85	0.706	0.294	0.000	0.853	0.147	2.527	0.180	21.1
TLR5	g.1736C>T	60	0.417	0.400	0.183	0.617	0.383	1.422	0.940	152.4
TLR5	g.3714C>T	77	0.273	0.494	0.234	0.519	0.481	0.010	0.500	96.3
TLR5	g.3891C>T	86	0.814	0.186	0.000	0.907	0.093	0.905	0.950	104.7
TLR5	g.4626T>C	63	0.095	0.429	0.476	0.310	0.690	0.000	0.700	226.2

^a^ SNP positions were determined in the reference sequences AC000135.1 and EU006635 reference sequences for *TLR4* and *TLR5*, respectively. ^b^ Value χ^2^ is not consistent with the Hardy–Weinberg equilibrium at *p* < 005. ^c^ Mean value of the read frequencies from the two NGS technologies, partially published by Novák et al. [16].

## Data Availability

The estimated breeding values for the bulls in this study are available from the Plemdat database at https://plm.eskot.cz (accessed on 19 December 2023) with permission from the Breeding Company CHD Impuls.

## Supplementary Materials

The following are available online at https://www.mdpi.com/article/10.3390/genes15010024/s1, Supplementary Table S1: Amplification primers for bovine genes *TLR4* and *TLR5*; Supplementary Table S2: Average breeding values for genotypic classes and significance of differences according to a *t*-test.
