# Supplementary material for: Association of Variants in Innate Immune Genes TLR4 and TLR5 with Reproductive and Milk Production Traits in Czech Simmental Cattle
Source: Genes (Basel). 2023 Dec 23;15(1):24. doi: 10.3390/genes15010024 (PMC10815032; doi:10.3390/genes15010024)
Supplement: Supplementary file 1 [file genes-15-00024-s001.zip › genes-2735449-supplementary/Novak et al. - Supplementary table S1.pdf]

Manuscript: "Association of variants in innate immune genes *TLR4* and *TLR5* with reproductive and milk production traits in Czech Simmental cattle" by Karel Novák, Terezie Valčíková, Kalifa Samaké and Marek Bjelka

Supplementary Table S1. Amplification primers for bovine *TLR4* and *TLR5* genes in the set of amplification reactions.

Gene	Reference sequence	Fragment denotation	Amplicon start	Amplicon end	Product length (base pairs)	Forward primer denotation	Forward primer sequence 5'→3'	Reverse primer denotation	Reverse primer sequence 5'→3'	Annealing temperature in PCR (°C)
TLR4	AC000135.1	T4_1	-3	657	661	4_1F	CCAGGGTATTTTGTTATGGCTGGAACAT	4_1R	TGTTTGCAAATGAACCTAACCA	62/60
TLR4	AC000135.1	T4_2	4999	5382	384	4_2F	TCTTTGCTCGTCCCAGTAGC	4_2R	AAGTGAATGAAAAGGAGACCTCA	62/60
TLR4	AC000135.1	T4_3	7941	9154	1214	4_3F	GGAGACCTAGATGACTGGGTTG	4_3R	AAGACAATGCGGATGTTGGT	62/60
TLR4	AC000135.1	T4_4	8924	9596	673	4_4F	TTTCAAGGGGTGCTGTTCTC	4_4R	TGCACACATCATTTGCTCAG	64/62
TLR4	AC000135.1	T4_5	9299	10110	812	4_5F	AGCCCAGACAGCATTTCAC	4_5R	CTATAGGGCTCGCGTACCAC	62/60
TLR4	AC000135.1	T4_6	9684	10420	737	4_6F	GTCACTGTGCTCCTGGTGTC	4_6R	GCCGCAGGAGAGACTTCT	64/62
TLR5	EU006635	T5_1	-3	638	642	5_1F	TTTGGGAAACGGAGGATAAG	5_1R	GCACCTTTGAGGCTGTGA	62/60
TLR5	EU006635	T5_2	553	1241	689	5_2F	GCCTGCTTTTGATACTTTGG	5_2R	AGGTGTCCGCTATGTTCTCA	62/60
TLR5	EU006635	T5_3	1065	1627	563	5_3F	TCCCTTACCTTCCAGCAGA	5_3R	AAGTTGGGGAAAACATTAGG	60/58
TLR5	EU006635	T5_4	1495	2036	542	5_4F	GGCAGATTAGAGGGGAAAGA	5_4R	CCATCAAAGAAGCAGGAAGA	58/56
TLR5	EU006635	T5_5	1927	2613	687	5_5F	TCACTCTCCCTTCTTCTCCA	5_5R	CAGACACTTGTTCCAGTCCA	60/58
TLR5	EU006635	T5_6	2529	3231	703	5_6F	CCTCCAAGGGAAAACACTCT	5_6R	ATTGGCTGTAAGTGGGATGT	60/58
TLR5	EU006635	T5_7	3153	3804	652	5_7F	TTTTCTTCCAAGCATTCCTA	5_7R	AGCCAGAGAGTTTGGGTACA	60/58
TLR5	EU006635	T5_8	3623	4195	573	5_8F	GAAACCAGCTCCTCTCTCCT	5_8R	ATCTTTCTGCTGCTCCACAC	62/60
TLR5	EU006635	T5_9	4059	4599	541	5_9F	AGACTTTGAATGGGTGCAGA	5_9R	TGGTAACTGGCGGAAATAAA	60/58
TLR5	EU006635	T5_10	4536	5299	764	5_10F	GGAGCAGTTTCCACTTATCG	5_10R	ATTCTCATGCCGGTTTCTTT	58/56
