# Supplementary material for: Association of Variants in Innate Immune Genes TLR4 and TLR5 with Reproductive and Milk Production Traits in Czech Simmental Cattle
Source: Genes (Basel). 2023 Dec 23;15(1):24. doi: 10.3390/genes15010024 (PMC10815032; doi:10.3390/genes15010024)
Supplement: Supplementary file 1 [file genes-15-00024-s001.zip › genes-2735449-supplementary/Novak et al. - Supplementary table S2.pdf]

Manuscript: "Association of variants in innate immune genes *TLR4* and *TLR5* with reproductive and milk production traits in Czech Simmental cattle" by Karel Novák, Terezie Valčíková, Kalifa Samaké and Marek Bjelka

Supplementary Table S2. Average breeding values for genotype classes and significance of differences according to the *t*-test.

Gene	SNP position[a]	Genotype	Milk production traits[b, c]					Udder health traits[b, c]				Reproductive traits[b, c]					
			FP (%)	FY (kg)	PP (%)	PY (kg)	MY (kg)	SCS	UHI	MLA	PER	CYS	ERD	CE	CM	PL	CVI
TLR4	245	GG	0.013	-5.86	0.027	-3.43	-155.43	98.00	96.71	98.43	95.43	99.14	100.71	101.71	96.29	96.14	98.71
TLR4	245	CG	0.010	1.71	0.017	2.12	26.92	97.61	98.33	98.86	99.45	98.87	100.92	103.06	98.06	97.88	102.12
TLR4	245	CC	0.024	-5.00	0.025	-3.65	-50.43	99.39	99.48	95.43	100.09	100.86	100.95	102.13	97.78	100.13	103.30
TLR4	610	CC	0.016	1.90	0.018	2.02	46.11	98.87	99.29	98.08	99.05	99.15	101.13	102.87	**98.48**^{a}	98.79	**101.13**^{a}
TLR4	610	CT	0.017	2.50	0.030	3.30	35.90	98.30	98.50	102.30	101.70	101.20	101.44	102.80	**93.30**^{b}	98.6	**105.60**^{b}
TLR4	5087	AA	0.015	0.33^{ab}	0.018	0.67	27.22	99.38	99.33	96.54	99.08	99.41	102.10	104.50	97.5	99.13	101.33
TLR4	5087	AG	0.040	0.69^{a}	0.019	-0.44	-48.53	98.81	99.25	98.50	99.19	98.47	100.86	102.94	97.75	97.56	101.81
TLR4	5087	GG	0.002	10.69^{b}	0.029	-6.77	-145.92	95.38	95.69	96.69	98.08	102.83	101.11	101.23	96.77	97.54	104.62
TLR4	5134	GG	0.021	-1.11	0.022	-0.58	-31.67	98.20	98.62	97.89	98.67	99.83^{a}	101.85	103.73	97.71	98.56	102.67
TLR4	5134	AG	0.033	-3.11	0.015	-3.37	-76.78	98.58	98.42	95.89	99.63	98.81^{ab}	100.23	100.84	98.37	96.37	100.53
TLR4	5134	AA	-0.005	5.00	-0.025	3.00	126.50	97.00	95.50	98.50	102.00	91.00^{b}	96.00	99.50	99.00	90.00	95.00
TLR4	7999	AA	0.032	-1.85	0.023	-1.54	24.67	97.92	98.69	96.54	103.62	101.46	100.10	103.08	97.54	**100.69**^{ab}	103.77
TLR4	7999	AG	0.023	0.25	0.021	0.31	-30.48	98.33	98.46	98.69	97.81	98.76	101.03	102.10	96.88	**96.54**^{a}	101.13
TLR4	7999	GG	-0.032	3.84	0.007	5.84	153.42	100.63	101.47	100.00	100.47	99.87	101.27	104.58	98.63	**103.32**^{b}	103.89
TLR4	9422	CC	0.033	6.33	0.018	4.58	94.25	98.67	98.50	99.42	99.67	**98.75**^{ab}	**99.36**^{ab}	101.92	**95.25**^{a}	**96.33**^{ab}	101.08
TLR4	9422	CT	0.004	-1.61	0.015	-0.39	-38.52	96.74	96.96	98.57	99.35	**96.75**^{a}	**98.33**^{a}	103.91	**96.83**^{ab}	**94.00**^{a}	101.22
TLR4	9422	TT	0.021	-3.71	0.022	-2.77	-39.93	98.87	99.42	97.45	101.65	**101.11**^{b}	**102.46**^{b}	101.84	**99.48**^{b}	**101.42**^{b}	102.39
TLR4	10310	TT	0.017	-0.70	0.020	-0.12	1.64	98.42	98.95	98.39	98.42	99.56	**101.98**^{a}	103.35	97.39	98.72	102.88
TLR4	10310	GT	0.020	-1.07	0.012	-1.04	-53.26	100.22	99.81	96.89	100.26	97.67	**98.45**^{b}	102.11	97.81	98.07	100.74
TLR5	488	CC	0.005	**10.20**^{a}	0.023	**10.27**^{a}	**243.33**^{a}	98.87	100.27	101.20	104.07	98.92	100.00	101.53	97.93	101.93	99.87
TLR5	488	CG	0.028	**2.03**^{b}	0.023	**1.66**^{b}	**92.44**^{b}	99.34	99.44	97.63	97.06	99.03	101.04	102.53	98.22	97.34	103.28
TLR5	488	GG	-0.001	**4.53**^{b}	0.015	**2.56**^{b}	**29.31**^{ab}	100.03	100.35	96.97	99.24	100.74	101.61	104.65	96.79	100.38	102.85
TLR5	545	TT	0.026	2.17	0.029	2.48	111.52	98.17	98.66	98.66	99.83	**101.26**^{ab}	102.36^{ab}	102.41	98.66	99.31	102.9
TLR5	545	CT	0.031	0.32	0.010	-0.76	-44.20	100.72	100.88	98.32	96.92	**97.28**^{a}	99.43^{a}	103.08	97.04	97.60	103.16
TLR5	545	CC	-0.006	-5.06	0.030	-1.63	-105.31	97.81	98.19	97.13	101.06	**98.50**^{b}	100.20^{b}	102.75	97.75	100.31	100.25
TLR5	619	GT	-0.005	-5.16	0.019	-2.48	-110.40	98.64	98.48	97.80	99.04	99.30	100.74	102.12	97.4	97.20	102.6

TLR5	619	TT	-0.003	-0.45	0.009	0.53	16.58	99.17	99.72	97.93	99.83	99.17	101.25	103.63	97.58	100.17	101.97
TLR5	1736	CC	-0.006	-0.04	-0.002	0.32	**12.64**[a]	97.88	97.72	96.20	100.68	98.50	101.68	102.92	**99.84**[a]	98.00	102.68
TLR5	1736	CT	0.009	-8.67	0.030	-5.58	**219.71**[b]	97.33	97.71	97.63	99.42	99.26	101.22	102.71	**96.08**[b]	97.00	102.04
TLR5	1736	TT	-0.025	3.00	-0.018	2.91	**240.20**[a]	99.45	100.36	99.36	100.00	98.70	100.00	104.18	**97.00**[ab]	101.36	103.09
TLR5	3714	CC	0.032	0.29	0.030	0.57	7.65	101.43	101.86	97.43	99.81	**102.50**[a]	**103.53**[a]	102.43	100.05[a]	100.19	102.10
TLR5	3714	CT	0.019	-0.32	0.018	0.03	2.14	99.16	99.55	97.82	97.11	**99.56**[ab]	**101.13**[ab]	103.47	97.18[b]	98.42	103.39
TLR5	3714	TT	0.009	-4.50	0.023	-2.61	-120.00	98.39	98.61	97.83	101.61	**97.40**[b]	**99.27**[b]	103.44	96.17[b]	99.11	101.67
TLR5	3891	CC	0.019	-2.06	0.019	**-1.41**[a]	-42.81	99.03	99.14	97.74	98.76	99.42	101.04	103.64	97.40	98.49	102.89
TLR5	3891	CT	-0.003	4.63	0.022	**5.94**[b]	123.88	98.56	99.75	99.06	102.56	100.43	101.23	101.44	98.69	101.19	101.06
TLR5	4626	TT	0.003	-8.17	-0.018	-8.17	-193.33	95.83	95.33	98.00	97.67	100.83	**104.83**[a]	102.00	98.50	95.33	98.83
TLR5	4626	CT	0.015	3.63	0.016	3.37	65.19	98.89	99.04	98.48	99.70	99.61	**102.45**[ab]	101.48	98.22	98.07	102.89
TLR5	4626	CC	0.022	-5.77	0.028	-4.17	-144.93	98.57	99.07	96.93	100.60	98.79	**99.84**[b]	104.53	96.20	99.50	101.60

[a] SNP positions were determined in the AC000135.1 and EU006635 reference sequences for *TLR4* and *TLR5*, respectively.

[b] Trait abbreviations: FP, milk fat percentage; FY, milk fat yield; PP, milk protein percentage; PY, milk protein yield; MY, milk yield; SCS, somatic cell score; UHI, udder health index; MLA, milkability; PER, lactation persistency, incidence of cystic ovaries (CYS), early reproductive disorders (ERD), calving ease (CE), maternal calving ease (CM), length of productive life (PL), and calf vitality index (CVI).

[c] Combinations showing significant differences in mean values are in bold. Values sharing the same letter index are not significantly different.
